# Metabolomics study of *Angelica sinensis* (Oliv.) Diels on the abnormal uterine bleeding rats by ultra‐performance liquid chromatography–quadrupole–time‐of‐flight mass spectrometry analysis

**DOI:** 10.1002/fsn3.2605

**Published:** 2021-10-13

**Authors:** Ting‐Ting Chen, Liang Zou, Di Wang, Wei Li, Yong Yang, Xiao‐Min Liu, Xin Cao, Jia‐Rong Chen, Yan Zhang, Jia Fu

**Affiliations:** ^1^ Affiliated Hospital of Chengdu University Chengdu University Chengdu China; ^2^ School of Pharmacy Dali University Dali China; ^3^ Key Laboratory of Coarse Cereal Processing of Ministry of Agriculture and Rural Affairs School of Food and Biological Engineering Chengdu University Chengdu China; ^4^ School of Pharmacy Chengdu University of Traditional Chinese Medicine Chengdu China; ^5^ School of Preclinical Medicine Chengdu University Chengdu China

**Keywords:** abnormal uterine bleeding, Angelica water extract, metabolomics, UPLC‐Q‐TOF‐MS

## Abstract

The objective of this study was to explore the effects and underlying intervention mechanisms of *Angelica* water extract (AWE) on abnormal uterine bleeding (AUB) based on serum metabolomics. Firstly, the concentration of main active substances in AWE was determined and the chemical components were identified by UPLC‐Q‐Exactive Orbitrap‐MS/MS. A drug‐induced abortion model was established by mifepristone and misoprostol. After administration AWE (2.16 g/kg) for 7 days, the coagulation function, serum hormone levels, H&E staining, and immunohistochemistry observation of uterus were detected. In addition, serum metabolites profiles were performed on ultra‐performance liquid chromatography–quadrupole–time‐of‐flight mass spectrometry (UPLC‐Q‐TOF‐MS). The contents of ferulic acid, senkyunolide A, and ligustilide in AWE were 0.7276, 0.0868, and 1.9908 mg/g, respectively. Twenty‐six compounds were identified in AWE. It was found that AWE was effective in regulation of coagulation function and promoting endometrial recovery. Meanwhile, the levels of E_2_, Pg, and HCG and the expression of ERα, Erβ, and PR were down‐regulated in AUB model and up‐regulated by the treatment of AWE. Twenty‐one potential biomarkers were eventually identified by multivariate statistical analysis. Study indicated that glycerophospholipid, sphingolipid, amino acids, retinol metabolism and primary bile acid biosynthesis were the main related metabolic pathways involved for the treatment of AUB by AWE. The results showed that AWE has potential therapeutic effect on AUB by altering the metabolic aberrations.

## Graphical abstract

Angelica water extract could reduce alleviate pathological injury and regulate the coagulation function to promote recovery of the endometrium. Angelica water extract could increase the levels of E2, Pg, and HCG and the expression of ERα, Erβ, and PR. Angelica water extract could regulate the metabolic pathways which include the glycerophospholipid metabolism, sphingolipid metabolism, glycine, serine and threonine metabolism, and retinol metabolism.

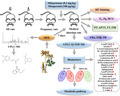

## 1 INTRODUCTION

In the early 1970s and 1980s, medical abortion became an alternative way for early termination of pregnancy (Regina et al., 2011). Mifepristone combined with misoprostol is the preferred clinical approach for the induction of abortion (Klaira & Paul, 2020), but the severe side effects of incomplete medical abortion still reached 15% (Ma et al., 2016). Abnormal uterine bleeding (AUB) is a common characteristic in incomplete medical abortion, with an incidence of approximately 3%–30% among reproductive‐aged women (Munro et al., 2018). The use of estrogen, tranexamic acid, multi‐dose compound contraceptive, and multi‐dose progesterone regimen are common clinically available non‐surgical options for AUB (Bradley & Gueye, 2015), but these treatments may cause multiple side effects (Yujie et al., 2018).

*Angelica sinensis* (Oliv.) Diels (Danggui) was widely used as a functional food or a dietary supplement in Asia, Europe, and America. It is also a famous traditional Chinese medicine for the treatment of anemia, dysmenorrhea, premenstrual, menopausal syndrome, and other gynecological diseases (Ma et al., 2015). Previous research indicates that polysaccharides, volatile oils, and organic acids are the main bioactive ingredients of *A*. *sinensis*. (Jin et al., 2016; Wei et al. 2016). The results of pharmacological studies indicated that *A*. *sinensis* can replenish and invigorate blood, prevent pain, and moisten the intestines (Ma et al., 2016). In addition, *A*. *sinensis* also have anti‐arrhythmic effects, enhanced immune function, cardioprotective effects, anti‐atherosclerotic effects, and inhibiting platelet aggregation (Gu et al., 2016). However, few reports focus on the underlying therapeutic effects and mechanisms of *A*. *sinensis* for AUB.

Metabolomics is a technique to study the metabolites and their dynamic changes before and after being stimulated or disturbed for the biological system (e.g., after a specific gene variation or environmental change). Metabolomics has been widely used in plant molecular phenotype (Showkat et al., 2019), drug safety (Chen et al., 2021; Yu et al., 2018), molecular pathology (Yang & Lao, 2019), mechanism of drug action (Su et al., 2020), and disease diagnosis (Karakioulaki & Stolz, 2019). In addition, it was also used to evaluate the impact of storage environment on food quality (Guo et al., 2019). Metabolomics is considered to be a useful strategy to explain the underlying mechanisms of TCM for the treatment of diseases. It emphasizes the study objects (humans or animals) as a unified whole, which is in accordance with the principle of integrity and dynamics of Traditional Chinese Medicine (TCM). The analysis methods of metabolomics mainly include nuclear magnetic resonance spectroscopy (NMR), gas chromatography‐mass spectrometry (GC‐MS), and liquid chromatography‐mass spectrometry (LC‐MS). Among all of the analytical technologies, UPLC‐Q‐TOF‐MS is becoming a key technology in biomarker discovery (Gika et al., 2014).

In the present study, the concentration of main active substances in AWE was determined and the chemical components were identified by UPLC‐Q‐Exactive Orbitrap‐MS/MS. The coagulation function, serum hormone levels, H&E staining, and immunohistochemistry observation of uterus were detected after the intervention of AWE. An UPLC‐Q‐TOF‐MS method and multivariate analysis were applied to identify the potentially authentic biomarkers. The purpose of this study was to reveal the underlying mechanisms of AWE for the treatment of AUB and to provide a theoretical basis for clinical application.

## 2 MATERIALS AND METHODS

### 2.1 Chemicals and reagents

*Angelica sinensis* was harvested in Longxi County, Gansu Province in July 2019, and identified by Prof. Ying Liu (School of preclinical medicine, Chengdu University). Ferulic acid (batch No. MUST‐20060511) was brought from Chengdu Mansite Biotechnology Co., Ltd. Senkyunolide A (batch No. wkq20050703) was brought from Sichuan Weikeqi Co., Ltd. Ligustilide (batch No. G01001909022) was brought from Chengdu Ruifensi Biotechnology Co., Ltd. Mifepristone and Misoprostol were brought from Zizhu Pharmaceutical Co. (Peking, China). Pg ELISA kit (batch No. VE3AZJHQ1W) and E_2_ ELISA kit (batch No. 44IBV1FHE5) were brought from Elabscience Biotechnology Co., Ltd (Wuhan, China). HCG ELISA kit (batch No. 11/2019) was brought from Shanghai MLBIO Biotechnology Co., Ltd. (Shanghai, China). Antibody against ERα (batch No. 00,046,360), Antibody against ERβ (batch No. 00,053,760), and Antibody against PR (batch No. 00,235,360) were brought from Abcam Co., Ltd. (Shanghai, China). HRP (batch No. 20,200,528) was brought from Bioss Co., Ltd. (Peking, China). PBS (batch No. 20,190,307) was brought from Zsbio Co., Ltd. (Peking, China). DAB (batch No. 07,062,019) was brought from Baso Co., Ltd. (Zhuhai, China).

### 2.2 Preparation of the water extract of *Angelica sinensis*

The water extract of *A*. *sinensis* (AWE) was extracted by heating reflux method. Thirty grams of crude herbal drugs was added to purified water ten times and extracted for twice (v/w), each extraction time was 30 min. The concentration of AWE was 0.6 g/ml after filtration (expressed by the weight per mL of crude drugs).

### 2.3 UPLC‐MS analysis of AWE

The analysis was performed in a Thermo Fisher Vanquish UPLC system with a Thermo Fisher Q Exactive (Iowa, USA). The mobile phase consisted of 0.1% formic acid in water (A) and acetonitrile (B). The elution program was as follows: 0–35 min, 95%–5% A; 35–35.01 min, 5%–95% A; and 35.01–40 min, 5% A. C18 column (4.6 × 100 mm, 2.7 µm) was maintained with the temperature of 30℃; flow rate, 0.4 ml/min; injection volume, 1 µl.

The MS operating parameters were as follows: the ion mode was positive; ion spray voltages, 3.5 kV; turbo spray temperature, 320℃; and m/z range, 100–1000. The main chemical constituents of *A*. *sinensis* were identified according to the exact molecular mass, the cleavage fragments of MS2, the mz cloud, mzVault 2.0 MS database, and literature review.

### 2.4 Determination of ferulic acid, senkyunolide A, and ligustilide in AWE by UPLC‐MS

The AWE was mixed with 50% methanol (1:1) and filtered through a 0.22 µm membrane filter. The concentration of AWE was 0.022 g/ml after filtration (expressed by the weight per mL of crude drugs).

The analysis was performed using an Vanquish UPLC system with a TSQ Fortis triple quadrupole mass spectrometer (Thermo Fisher, USA), Accucore™ C18 column (2.1mm × 100mm, 2.6 µm, Thermo Fisher, USA). The mobile phase consisted of 0.1% formic acid in water (A) and acetonitrile (B). The UPLC elution program was as follows: 0–5 min, 85%‐60% A; 5–10 min, 60%‐55% A; 10–18 min, 55%‐30% A; 18–18.01 min, 30%‐85% A; and 18.01–23 min, 85% A, and Injection volume, 10 µl; flow rate, 0.2 ml/min; column temperature, 35 ℃. The Mass operating parameters were as follows: The ion mode was positive; scan type, SRM; sheath gas flow rate, 35 arb; aux gas flow rate, 15 arb; aux gas heater temp, 350℃; spray voltage, 3.5 kV; and capillary temp, 350℃.

### 2.5 Animal experiments

Female Sprague–Dawley (*SD*) rats of specific pathogen‐free (SPF) status, weighing 200–220 g; and male *SD* rats of SPF status, weighing 250–300 g (Certificate No. SCXK (Chuan) 2020‐030) were brought from the Chengdu Dossy Experimental Animals CO.LTD. (Chengdu, China). The all animals were kept under the same conditions. All experimental protocols were approved by the Animal Ethics Committee of the Chengdu University (20191209‐lxsz003).

The AUB rat model was established by mifepristone and misoprostol according to the method of previous literature (Zuo et al., 2019). The pregnancy control group (P) and AUB model group (M) were given with sterile saline, and the AUB + AWE group was administrated with dosage of 2.16 g/kg AWE once a day for 7 days.

### 2.6 Histopathological examination

The uterine tissues were immediately dissected after the experiment, removed fat and connective tissue, and fixed in 4% paraformaldehyde solution. Then, the uterine tissues were dehydrated at 4℃ for 24–48 hr, conventionally paraffin embedded, sectioned at 4 µm, and stained with hematoxylin–eosin (HE). Pathological changes of the endometrium were observed and photographed under a microscope.

### 2.7 Measurement of serum hormone levels

The serum levels of progesterone (Pg), estradiol (E_2_), and human chorionic gonadotrophin (HCG) were measured according to the instructions of manufacturer of the ELISA kits, respectively.

### 2.8 Detection of plasma coagulation function

Collected blood (3 ml) from the abdominal aorta with sodium citrate at a mass concentration of 3.8 g/L (anticoagulant: blood = 1:9) was centrifuged to obtain plasma. Prothrombin time (PT), thrombin time (TT), activated partial thrombin activity time (APTT), and fibrinogen (FIB) were determined by a hemagglutination analyzer.

### 2.9 Protein distribution analyses by immunohistochemistry

The uterine tissues were dehydrated, defatted, and conventionally paraffin embedded. Then, the uterine tissues were sectioned at 4 µm, deparaffinized, and rehydrated. After that, the tissue sections were incubated with 3% H_2_O_2_, repaired in antigen recovery solution, and then sealed at room temperature for 20 min after drip‐adding normal goat serum blocking solution. Then, the samples were incubated at 4 ℃ overnight with primary inhibitors (dilution 1:200): estrogen receptor α (ERα), estrogen receptor β (ERβ), and progesterone receptor (PR). One day after incubation, the cells were washed with PBS three times for 5 min each time, and then, drip‐added and incubated the secondary antibody at room temperature for 1 hr. SABC was added and incubated at 37℃ for 1 hr. Stained the proteins to dark‐brown by immersion in diaminobenzidine. The slices were rinsed with deionized water for 10 min. Hematoxylin was used for counterstaining, and hydrochloric acid alcohol was differentiated. Routine dehydration, transparentizing, sealing, and microscopy were performed.

### 2.10 Serum sample preparation

Added 400 µl anhydrous acetonitrile containing internal standard into 100 µl serum sample and vortex mixed for 3 min. Then, centrifuged the mixture at 12,000 rpm for 10 min to obtain the supernatant and putted it into a sampling vial.

### 2.11 UPLC‐Q‐TOF‐MS analysis conditions

An UPLC‐Q‐TOF‐MS system (Agilent, USA) was used for analysis with a BEH C18 column (2.1 mm × 100 mm, 1.7 µm). 0.1% formic acid in water (A) and acetonitrile (B) was used as a mobile phase with the following elution program: 0–3 min, 10%–30% B, 3–25 min, and 30%–95% B. Column temperature, 35℃; flow rate, 0.35 ml/min. The full scan range was 50 to 1,200 m/z; sheath gas temperature, 320℃; sheath gas flow, 12 L/min; drying gas temperature, 300℃; drying gas flow, 6 L/min; capillary voltage, 3.5 kV; and nebulizer pressure, 1.0 bar.

### 2.12 Data processing

The partial least‐squares discriminant analysis (PLS‐DA) and orthogonal partial least‐squares discriminant analysis (OPLS‐DA) were used for data analysis. The database used to identify the potential biomarkers was as follows: https://hmdb.ca/, http://www.lipidmaps.org/, http://www.genome.jp/kegg/, http://metlin.scripps.edu/. All results were described as the mean ± standard deviation (*SD*). One‐way analysis of variance (ANOVA) was used to analyze study data for significance comparison.

## 3 RESULTS

### 3.1 Identification of major compounds in AWE

**FIGURE 1:**
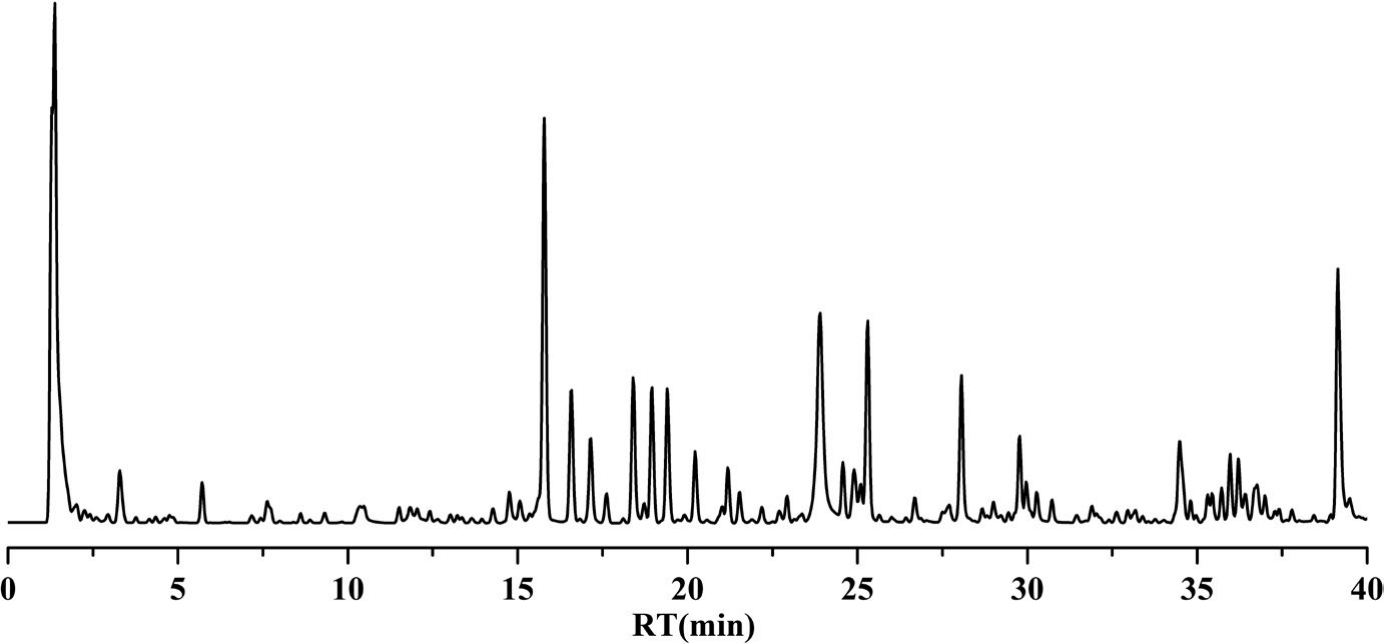
The total ion chromatograms of samples by UPLC‐MS

The representative chromatography was shown in Figure 1. Twenty‐six constituents were identified by the accurate mass and relative ion abundance of the target peaks. The main constituents in AWE were γ‐Aminobutyric acid (GABA), Nystose, l‐Valine, Nicotinic acid, 4‐Oxoproline, Guanosine, Succinic acid, l‐Phenylalanine, dl‐Tryptophan, 2‐Anisic acid, Isophthalic acid, Caffeic acid, l‐Histidine, N‐Acetyl‐d‐alloisoleucine, Vanillin, Isofraxidin, Ferulic acid, Azelaic acid, Coniferyl aldehyde, Berberine, Jatrorrhizine, Coptisine chloride, Palmatine, Ligustilide, Senkyunolide A, and Levistilide A. The area percentage of the constituents is shown in Table 1.

**TABLE 1:** The information of compounds identified in AWE by UPLC‐MS

Peak	Constituent	Formula	Molecular Weight	RT (min)	Area(Max.)	M/Z	Fragments
1	γ‐Aminobutyric acid (GABA)	C_4_H_9_NO_2_	103.0639	2.500	1.12E+09	104.0714	87.04505
2	Nystose	C_24_H_42_O_21_	666.2216	2.563	1.51E+08	711.2197	665.21375,485.15158,341.10703,221.06606,179.05527,89.02335
3	L‐Valine	C_5_H_11_NO_2_	117.0795	2.734	4.18E+08	118.0867	72.08173,55.05518
4	Nicotinic acid	C_6_H_5_NO_2_	123.0328	3.298	2.58E+08	124.0395	96.05189,80.05059
5	4‐Oxoproline	C_5_H_7_NO_3_	129.0417	3.427	3.28E+08	128.0345	82.02883
6	Guanosine	C_10_H_13_N_5_O_5_	283.0923	4.155	1.59E+09	284.0998	152.05748
7	Succinic acid	C_4_H_6_O_4_	118.0258	4.250	8.39E+07	117.0185	73.02842
8	L‐Phenylalanine	C_9_H_11_NO_2_	148.0530	5.404	1.48E+09	166.0869	120.08154,103.05509
9	DL‐Tryptophan	C_11_H_12_N_2_O_2_	204.0919	6.996	3.04E+09	188.0725	146.06157,118.06660
10	2‐Anisic acid	C_8_H_8_O_3_	152.0467	7.592	2.45E+08	151.0394	123.04420,107.04922,93.03354
11	Isophthalic acid	C_8_H_6_O_4_	166.0260	8.380	1.24E+09	165.0186	121.02848
12	Caffeic acid	C_9_H_8_O_4_	180.0420	9.202	8.76E+08	179.0347	135.04422,71.01277
13	L‐Histidine	C_6_H_9_N_3_O_2_	155.0688	9.876	7.35E+07	154.0616	137.03474,110.07134,93.04478
14	*N*‐Acetyl‐D‐alloisoleucine	C_8_H_15_NO_3_	173.1048	10.273	1.52E+07	172.0974	130.08641
15	Vanillin	C_8_H_8_O_3_	152.0467	11.346	9.69E+07	151.0394	136.01569
16	Isofraxidin	C_11_H_10_O_5_	222.0529	11.967	3.23E+07	221.0455	206.02150,190.99799,163.00291,135.00803
17	Ferulic acid	C_10_H_10_O_4_	194.0574	12.226	7.56E+07	193.0503	178.02640,134.03628
18	Azelaic acid	C_9_H_16_O_4_	188.1044	13.153	3.51E+08	187.0971	125.09621,97.06487
19	Coniferyl aldehyde	C_10_H_10_O_3_	178.0626	13.573	8.28E+07	177.0553	162.03142
20	Berberine	C_20_H_17_NO_4_	335.1177	13.627	4.55E+07	336.1250	320.09439,292.09912
21	Jatrorrhizine	C_20_H_19_NO_4_	337.1354	13.691	9.45E+08	338.1427	322.11090,308.09509,294.11551,280.09985
22	Coptisine chloride	C_19_H_14_ClNO_4_	319.0885	13.998	8.28E+07	320.0958	292.09979
23	Palmatine	C_21_H_21_NO_4_	351.1500	15.186	1.86E+08	352.1573	336.12640,308.13129
24	Ligustilide	C_12_H_14_O_2_	190.0996	22.968	1.80E+08	191.1065	173.09673,145.10168,117.07053,91.05499
25	Senkyunolide A	C_12_H_16_O_2_	192.1150	23.990	2.08E+08	193.1222	175.11226,147.11722,137.06015,105.07059,91.05498
26	Levistilide A	C_24_H_28_O_4_	380.1986	32.075	4.40E+08	381.2062	191.10716

### 3.2 Determination of ferulic acid, senkyunolide A, and ligustilide in AWE

**FIGURE 2:**
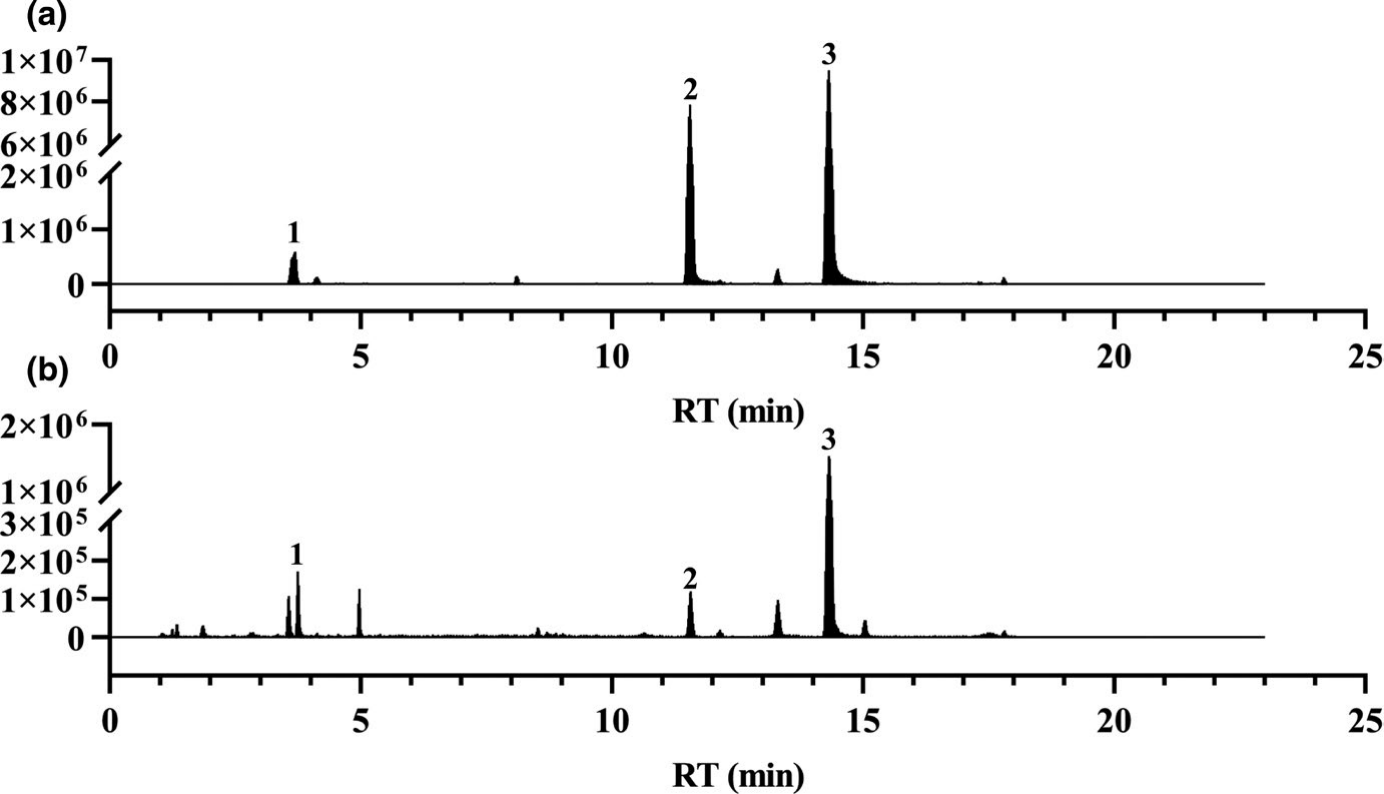
The chromatogram of mixed reference solution and AWE by UPLC‐MS. (a) Mixed reference solution, (b) AWE. 1. Ferulic acid, 2. Senkyunolide A, 3. Ligustilide

The representative chromatography was shown in Figure 2. Ferulic acid was linear over a concentration range of 3.10–154.90 µg/ml, senkyunolide A was linear at 1.33–40.00 µg/ml, and ligustilide was linear at 2.04–254.65 µg/ml. The concentration of the mixed reference solution was taken as the abscisic coordinate, and the peak area was taken as the ordinate for linear regression. Typical equation of calibration curve for ferulic acid was *y* = 14333x + 63,294 (*r* = 0.9970); the curve of senkyunolide A was *y *= 343389x−340450 (*r* = 0.9978); and that of ligustilide was *y* = 88,686x + 876,354 (*r* = 0.9953). The contents of ferulic acid, senkyunolide A, and ligustilide were 0.7276, 0.0868, and 1.9908 mg/g, respectively.

### 3.3 AWE improved the histopathological damage

**FIGURE 3:**
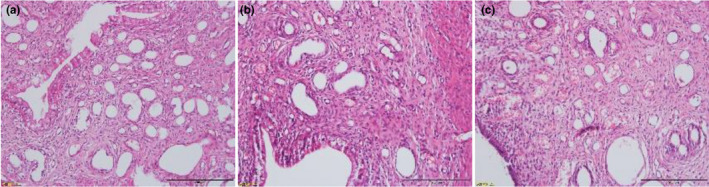
Effects of AWE on pathological changes of endometrium in rats by HE Staining (200×). (a) Pregnant group, (b) AUB model group, (c) AWE group

Microscopic examination explored that the endometrium of the P group was significantly thickened, the uterine cavity was small, the glands and blood vessels in the lamina propria were rich and dilated, and some of them were hyperemic (Figure 3a). Compared with the P group, the endometrium was thin, with local defects, and the lamina propria was mainly showed densely distributed blood vessels with slight congestion in the M group (Figure 3b). The endometrium in the AWE group was rich in blood vessels and loose in the stroma, but the symptoms were less severe than those in the model group (Figure 3c).

### 3.4 Measurement of serum hormone levels

**FIGURE 4:**
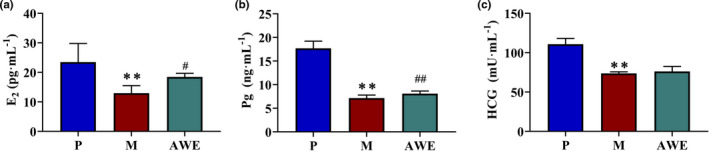
The E_2_, Pg, and HCG Levels in each group. (a) E_2_, (b) Pg, (c) HCG. The results were presented as the mean ± SD, *n* = 6. **p < .01 AUB model group versus pregnant group, #*p* < .05 AWE group versus AUB model group, ##*p* < .01 AWE group versus medical aborting group

Comparing with the P group, the E_2_, Pg, and HCG levels were significantly decreased in the M group (*p* < .01). The E_2_ and Pg levels were significantly increased with the treatment of AWE (*p* < .05) (Figure 4a–c).

### 3.5 Effects of AWE on blood coagulation function in rats

**FIGURE 5:**
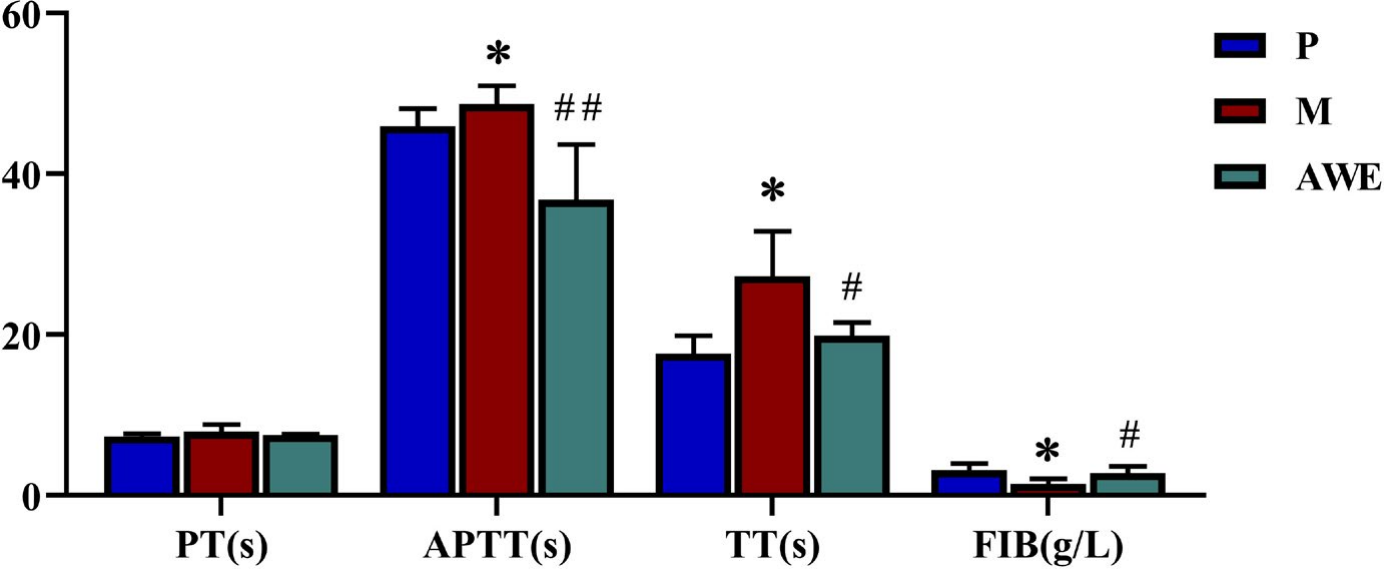
The four coagulation indices of each experimental group. The results were presented as the mean ± SD, *n* = 6. *p < .05 AUB model group versus pregnant group, #*p* < .05 AWE group versus AUB model group, ##*p* < .01 AWE group versus AUB model group

Compared with the P group, the APTT and TT levels were significantly longer and FIB level was significantly lower in the M group (*p* < .05). The APTT and TT levels were significantly lower, and FIB level was significantly longer with the treatment of AWE (*p* < .05). (Figure 5).

### 3.6 Effects of AWE on ERα, Erβ, and PR levels in rats

**FIGURE 6:**
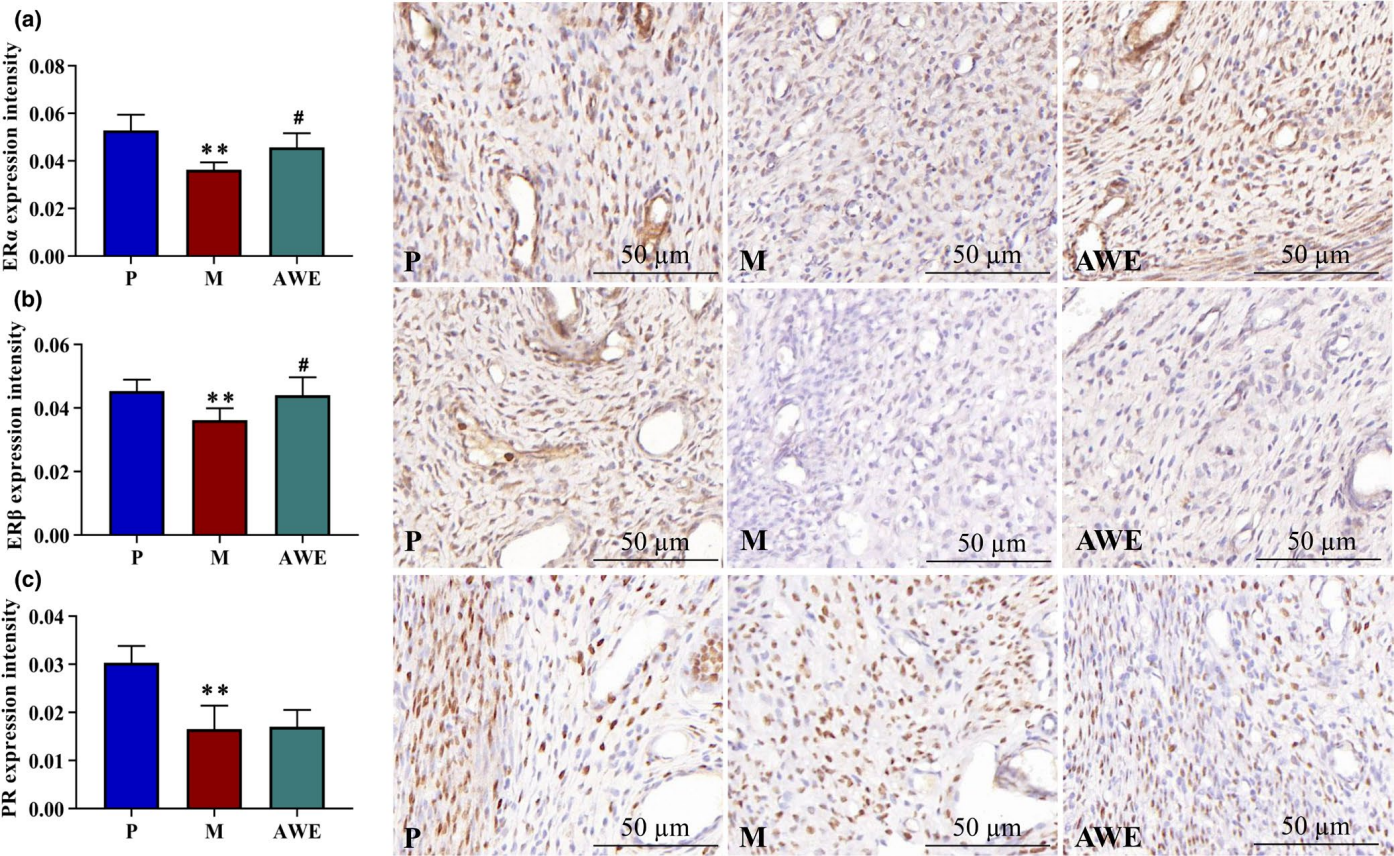
The ERα, Erβ, and PR Levels in each group. (a) ERα, (b) ERβ, (c) PR. The results were presented as the mean ± SD, *n* = 6. **p < .01 AUB model group versus pregnant group, #*p* < .05 AWE group versus AUB model group

Expression of ERα, Erβ, and PR was reduced in the M group when compared to the P group (*p* < .01). The expression of ERα and ERβ was increased with the treatment of AWE (*p* < .05). (Figure 6a–c).

### 3.7 Data quality assurance of UPLC‐Q‐TOF‐MS

**FIGURE 7:**
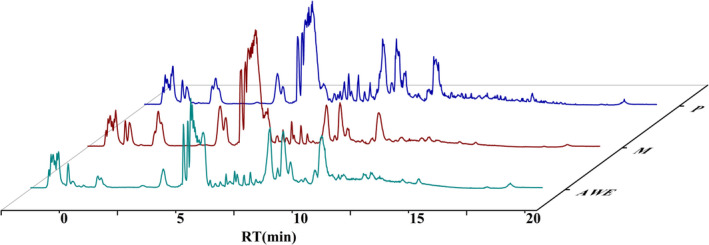
The typical total ion chromatography of all serum samples in positive mode. P: Pregnant group, M: AUB model group, AWE: AWE group

The typical total ion current chromatograms of each group of serum samples were shown in Figure 7.

### 3.8 Differential metabolites between the AUB and the pregnancy rats

**FIGURE 8:**
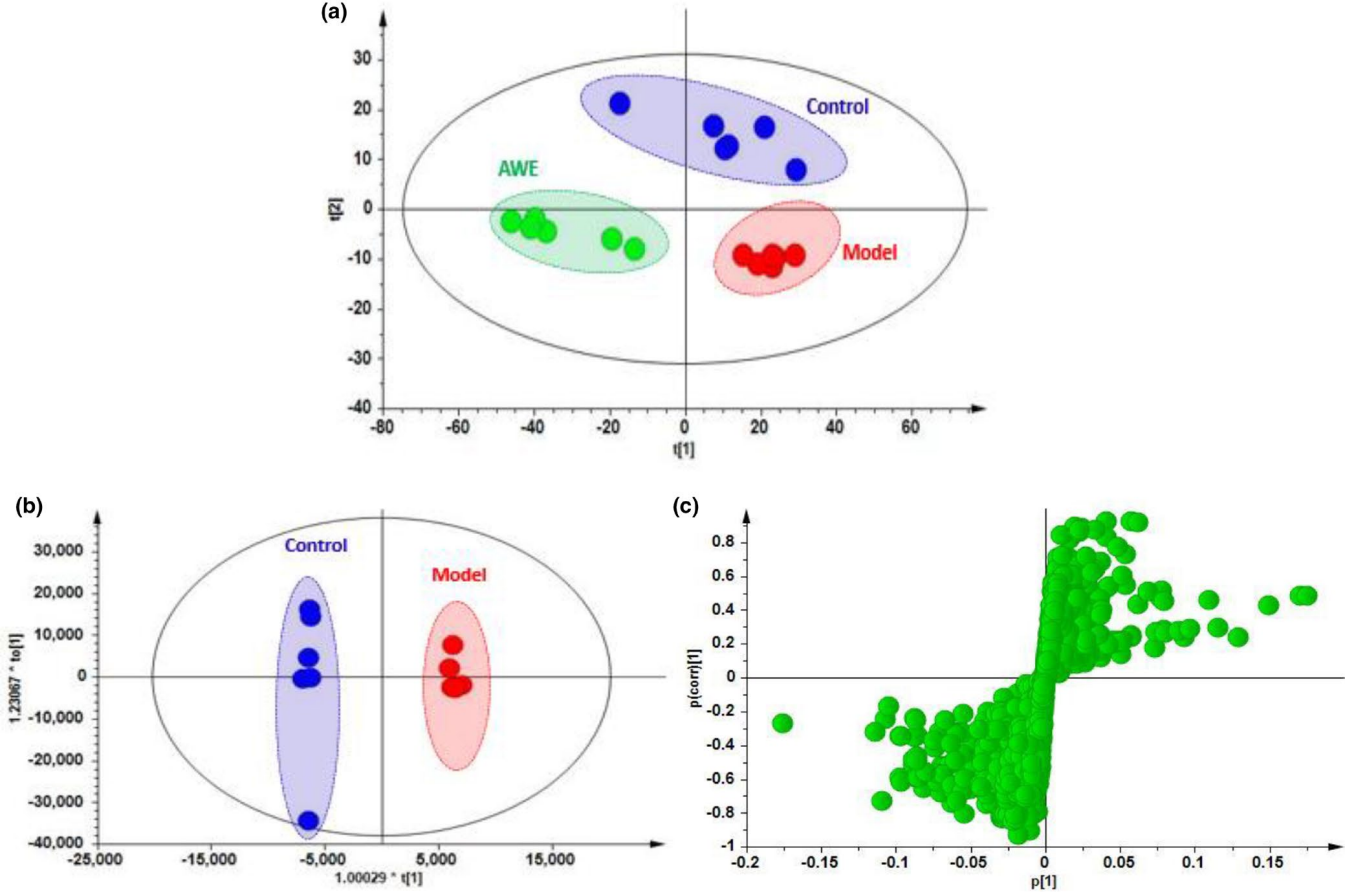
The multivariate statistical analysis. (a) PLS‐DA; (b) OPLS‐DA; (c) OPLS‐DA s‐plot

**FIGURE 9:**
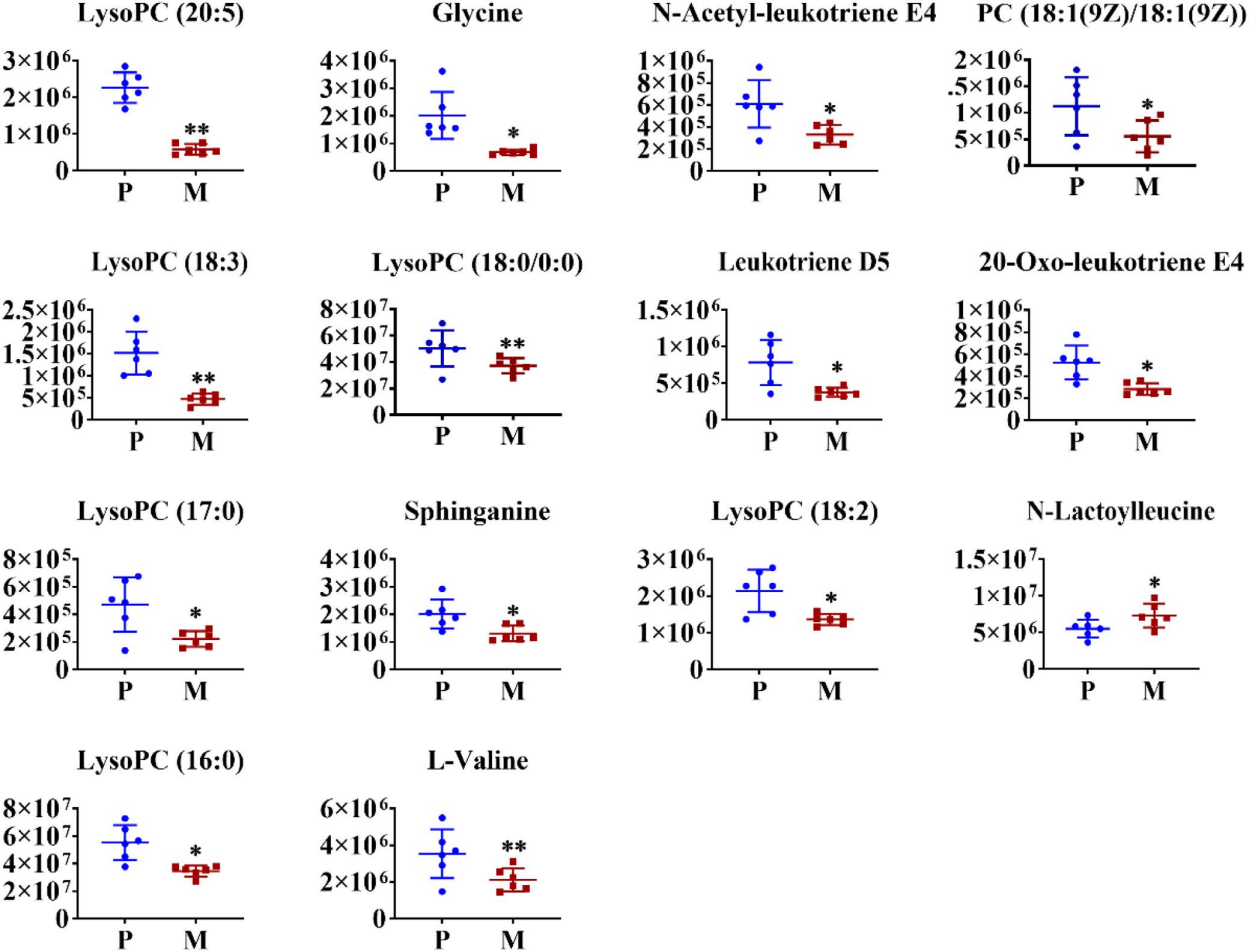
The relative intensities of the examined metabolites obtained from P and M groups. The results were presented as the mean ± SD, *n* = 6. *p < .05 versus pregnant group, ** *p* < .01 versus pregnant group

**FIGURE 10:**
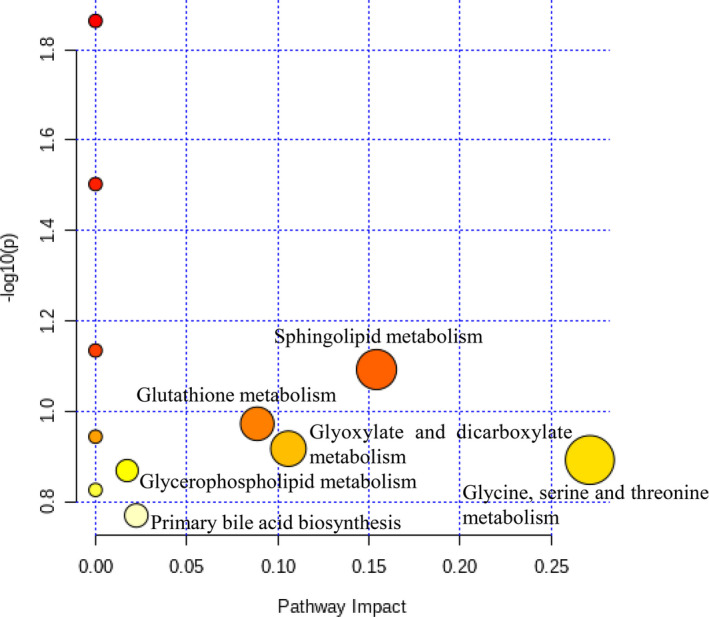
Summary of pathway analysis of P and M groups

The PLS‐DA and OPLS‐DA analyses explored that there was an obvious separation between the P, M, and AWE groups (Figure 8a,b). The S‐plots indicated the contribution of different metabolites variables between the P and M groups (Figure 8c). Fourteen significantly differential metabolites were shown in Table 2. LysoPC (20:5), Glycine, N‐Acetyl‐leukotriene E4, PC (18:1(9Z)/18:1(9Z)), LysoPC (18:3), LysoPC (18:0/0:0), Leukotriene D5, 20‐Oxo‐leukotriene E4, LysoPC (17:0), Sphinganine, LysoPC (18:2), LysoPC (16:0), L‐Valine, and N‐Lactoylleucine were significantly lower in the M group compared with the P group (Figure 9). The metabolism pathways of glycine, serine and threonine, glyoxylate and dicarboxylate, glycerophospholipid, primary bile acid biosynthesis, glutathione, and sphingolipid were significantly altered in the M group (Figure 10).

**TABLE 2:** 14 Differential metabolites in the serum of P and M groups

Compound	Formula	Metabolites	M/Z	Rt (min)	VIP
A1	C_28_H_48_NO_7_P	LysoPC (20:5)	542.3176	9.604	4.6232
A2	C_26_H_43_NO_9_S	Glycine	546.3472	10.749	3.5524
A3	C_25_H_39_NO_6_S	*N*‐Acetyl‐leukotriene E4	482.3185	11.836	1.7062
A4	C_44_H_84_NO_8_P	PC (18:1(9Z)/18:1(9Z))	786.5852	16.334	2.3486
A5	C_26_H_48_NO_7_P	LysoPC (18:3)	518.3189	9.676	3.8540
A6	C_26_H_54_NO_7_P	LysoPC (18:0/0:0)	524.3638	12.447	1.2658
A7	C_25_H_38_N_2_O_6_S	Leukotriene D5	495.3220	9.898	2.3342
A8	C_23_H_35_NO_6_S	20‐Oxo‐leukotriene E4	454.2870	10.300	1.2371
A9	C_25_H_52_NO_7_P	LysoPC (17:0)	510.3476	11.330	1.8070
A10	C_18_H_39_NO_2_	Sphinganine	302.2703	8.897	1.3561
A11	C_26_H_50_NO_7_P	LysoPC (18:2)	520.3303	11.049	3.1960
A12	C_24_H_50_NO_7_P	LysoPC (16:0)	496.3338	10.884	10.9197
A13	C_5_H_11_NO_2_	L‐Valine	118.0849	0.937	2.9892
A14	C_9_H_17_NO_4_	*N*‐Lactoylleucine	204.1198	1.003	5.7701

### 3.9 Differential metabolites between AUB and AWE treatment rats

**FIGURE 11:**
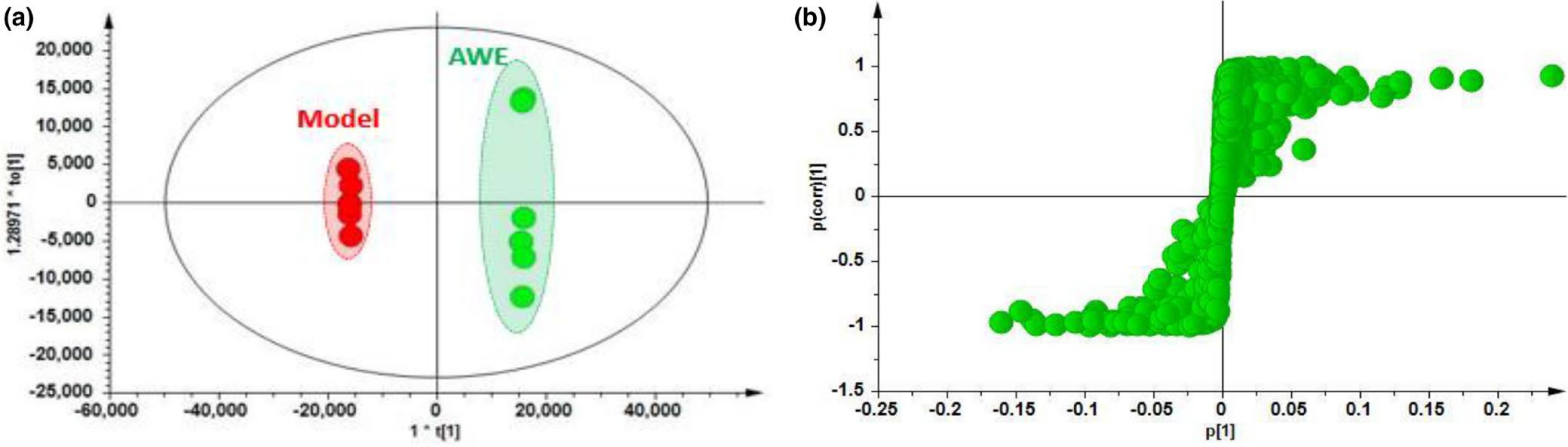
The results of multivariate statistical analysis from the serum samples. LC‐MS positive (a and b), (a) OPLS‐DA; (b) OPLS‐DA s‐plot

**FIGURE 12:**
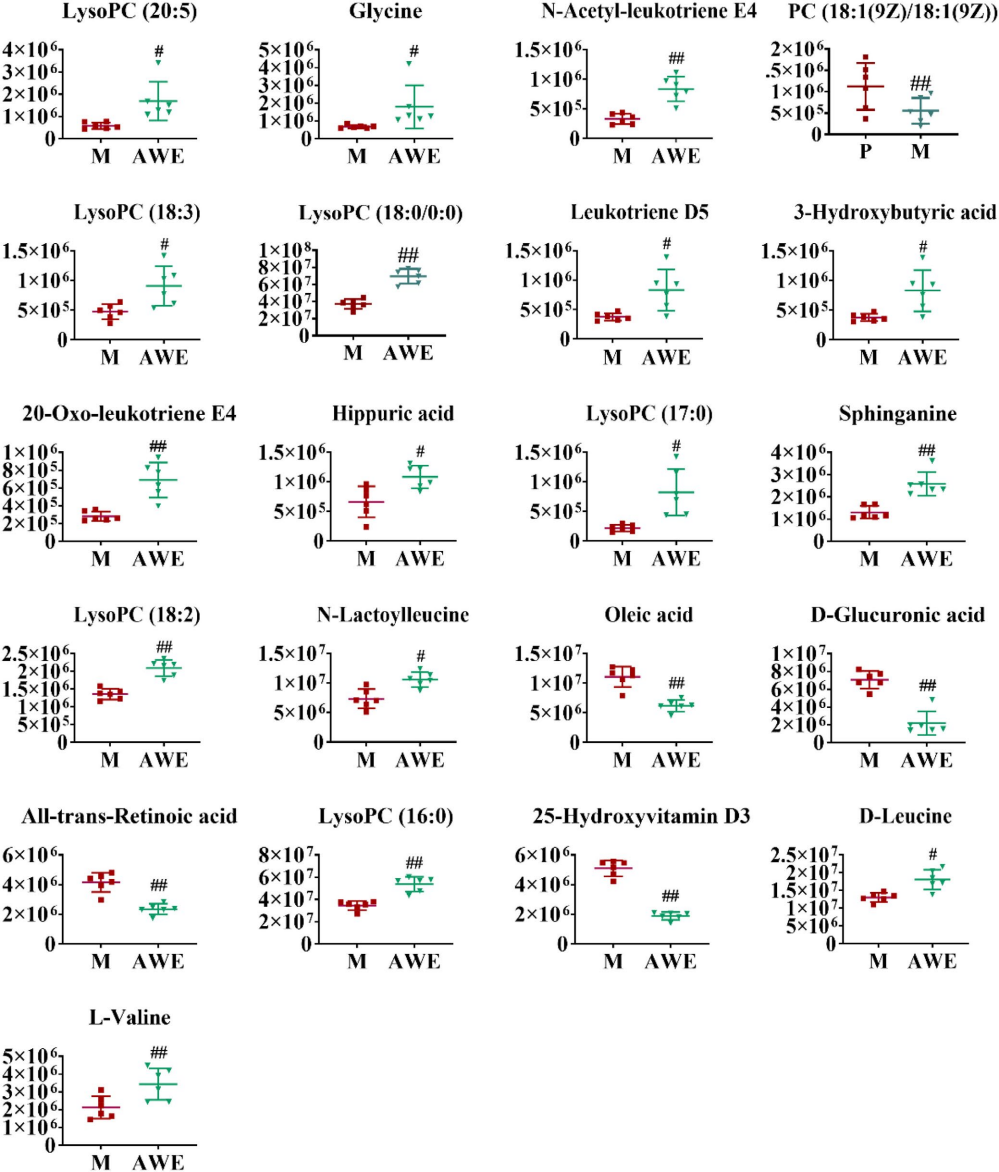
The relative intensities of the examined metabolites obtained from M and AWE groups. The results were presented as the mean ± SD, *n* = 6. #*p* < .05 versus. AUB model group, ## *p* < .01 versus AUB model group

**FIGURE 13:**
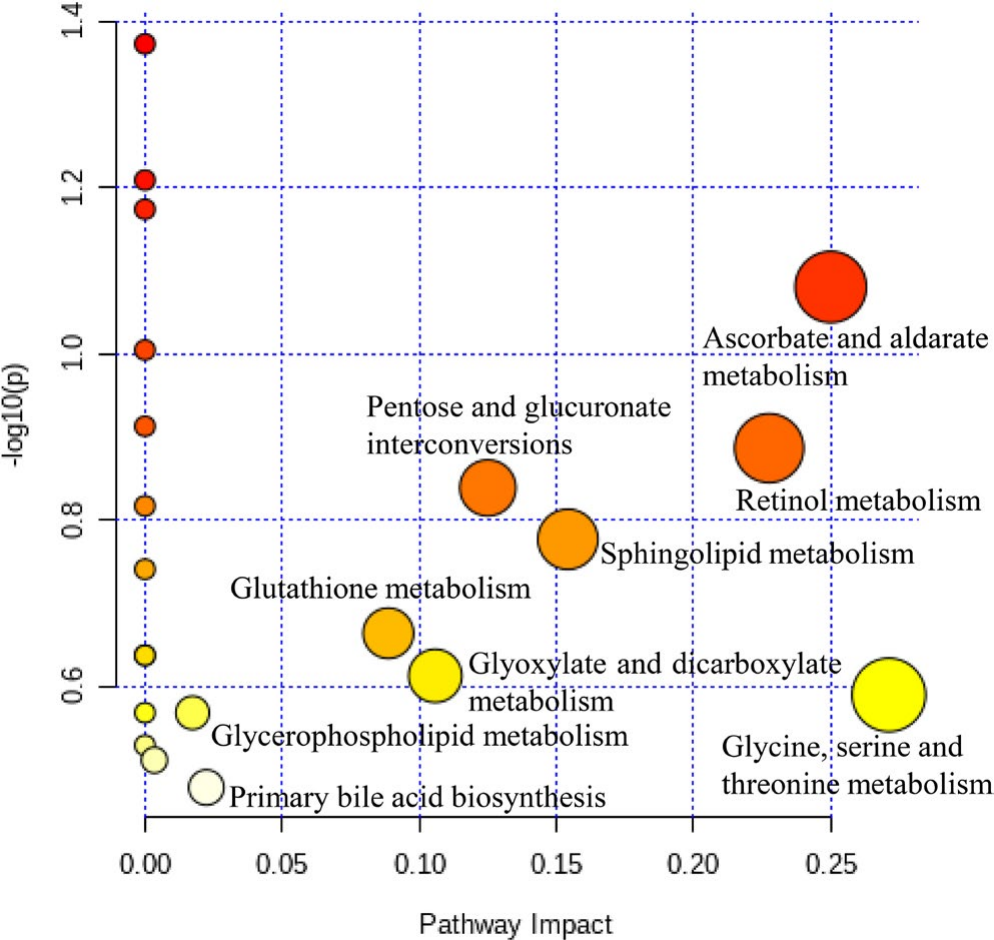
Summary of pathway analysis of M and AWE groups

There was a significant separation between the M and AWE groups in the OPLS‐DA model (Figure 11a), indicating that AWE had an effect on the metabolic profile of AUB rats. The S‐plots indicated the contribution of different metabolites variables between the M and AWE groups (Figure 11b). Twenty‐one significantly differential metabolites were shown in Table 3, and there showed that the specific changes of the relative content in these specific metabolites (Figure 12). LysoPC (20:5), Glycine, N‐Acetyl‐leukotriene E4, PC (18:1(9Z)/18:1(9Z)), LysoPC (18:3), LysoPC (18:0/0:00), Leukotriene D5, 3‐Hydroxybutyric acid, 20‐Oxo‐leukotriene E4, Hippuric acid, LysoPC (17:0), D‐Leucine, and L‐Valine were significantly higher in the AWE group compared with the M group. However, the other metabolites, including D‐Glucuronic acid, All‐trans‐Retinoic acid, and 25‐Hydroxyvitamin D3, were significantly lower in the AWE group compared with the M group. The metabolism pathways of primary bile acid biosynthesis, pentose and glucuronate interconversions, glycerophospholipid, glutathione, glyoxylate and dicarboxylate, sphingolipid, glycine, serine and threonine, retinol, ascorbate, and aldarate were significantly altered in the M group (Figure 13).

**TABLE 3:** 21 Differential metabolites in the serum of M and AWE groups

Compound	Formula	Metabolites	M/Z	Rt (min)	VIP
B1	C_28_H_48_NO_7_P	LysoPC (20:5)	542.3176	9.604	1.7704
B2	C_26_H_43_NO_9_S	Glycine	546.3472	10.749	1.6321
B3	C_25_H_39_NO_6_S	*N*‐Acetyl‐leukotriene E4	482.3185	11.836	1.3480
B4	C_44_H_84_NO_8_P	PC (18:1(9Z)/18:1(9Z))	785.5852	16.334	1.7397
B5	C_26_H_48_NO_7_P	LysoPC (18:3)	518.3189	9.676	1.1027
B6	C_26_H_54_NO_7_P	LysoPC (18:0/0:0)	524.3638	12.447	11.0291
B7	C_25_H_38_N_2_O_6_S	Leukotriene D5	495.3220	9.898	1.1269
B8	C_4_H_8_O_3_	3‐Hydroxybutyric acid	105.0338	4.681	1.0865
B9	C_23_H_35_NO_6_S	20‐Oxo‐leukotriene E4	454.2870	10.300	1.2019
B10	C_9_H_9_NO_3_	Hippuric acid	180.0650	4.681	1.2399
B11	C_25_H_52_NO_7_P	LysoPC(17:0)	510.3476	11.330	1.3592
B12	C_18_H_39_NO_2_	Sphinganine	302.2703	8.897	1.4056
B13	C_26_H_50_NO_7_P	LysoPC(18:2)	520.3303	11.049	2.0366
B14	C_9_H_17_NO_4_	*N*‐Lactoylleucine	204.1198	1.003	1.6609
B15	C_18_H_34_O_2_	Oleic acid	283.1748	5.656	2.7529
B16	C_6_H_10_O_7_	D‐Glucuronic acid	195.1221	1.815	4.0168
B17	C_20_H_28_O_2_	all‐trans‐Retinoic acid	301.2044	5.656	4.2104
B18	C_24_H_50_NO_7_P	LysoPC(16:0)	496.3338	10.884	2.4304
B19	C_27_H_44_O_2_	25‐Hydroxyvitamin D3	401.3184	7.223	8.3933
B20	C_6_H_13_NO_2_	D‐Leucine	132.1005	0.927	3.4918
B21	C_5_H_11_NO_2_	L‐Valine	118.0849	0.937	4.1642

## 4 DISCUSSION

*Angelica sinensis*, as a medicinal food, has widely been used for the treatment of amenorrhea, dysmenorrheal, and premenstrual syndrome of gynecological disorders (Li et al., 2012). Studies have shown *A*. *sinensis* contains ferulic acid, senkyunolide A, ligustilide, etc., so it was speculated that AWE had a certain influence on the anti‐inflammatory (Fang et al., 2020), blood replenishing (Tao et al., 2016), liver lipid accumulation, and fatty regeneration (Ma et al., 2020). Peng Cao et al. found *Angelica sinensis* polysaccharide as a kind of “tonic foods,” which has potential to be used as a hepatoprotective agent for Acetaminophen‐induced hepatic damage (Cao et al., 2018). Yong li Hua et al. found that *A*. *sinensis* can promote hematopoiesis, enhance antiapoptotic effects, and regulate energy metabolism (Hua et al., 2017). Qin Fan et al. found that ferulic acid could scavenge PPH‐ and ABTS‐free radicals, while ligustilide exhibited scavenging capacity for ABTS‐free radicals (Fan et al., 2020). Zi‐wen Yuan et al. found that *A*. *sinensis* intervention could significantly relieve blood stasis syndrome in rats (Yuan et al., 2019). In this study, APTT and TT levels were significantly lower in AWE group (*p* < .05), the APTT and TT levels were significantly lower, and FIB level was significantly longer with the treatment of AWE, indicating that *A*. sinensis can regulate blood coagulation function of AUB rats.

Metabolomics is a large‐scale research technology that uses modern analytical method to assess the creature physiological status in different conditions (Wei et al., 2020). The metabolic profiles reflect an individual's function state at a certain point, which is consistent with the integrality and systematicness of traditional Chinese medicine (Bao et al., 2017). This technique has been used to study the effects of Chinese medicine syndrome patterns (Fengxia et al., 2010; Li et al., 2016). As an important part of the human body, blood contains abundant information and is often used as the matrix for metabolomics research. Therefore, the UPLC‐Q‐TOF‐MS metabolomics platform and multivariate statistical analysis method were used to assess the rats’ serum to reveal the mechanism of AWE in AUB caused by incomplete abortion. In our study, we identified 21 potential biomarkers that were directly or indirectly correlated for the therapeutic effects of AWE in AUB caused by incomplete abortion, mainly including amino acids, retinol, fatty acids, and lysophospholipids. We have found that the serum of the incomplete medical abortion rats showed altered metabolism mainly in amino acid, retinol, lipid metabolism and primary bile acid biosynthesis pathways.

Amino acids are necessary for embryonic growth and development. In our study, we found that the level of some amino acids including glycine, d‐leucine, and l‐valine was abnormal in the AWE group. Glycine was closely associated with inflammation among these amino metabolites (Angélica et al., 2014). Glycine promotes myelin phagocytosis and the production NO and TNF‐α, which may affect immunological processes of inflammatory diseases (Carmans et al., 2010). In the inflammation, the TNF‐α induces protein decomposition and catabolism to up‐regulate the urea synthesis (Louise et al., 2013). Yong‐li Hua et al. found that volatile oil from *A*. *sinensis* can inhibit inflammation through down‐regulating the synthesis of glycine, arachidonic acid, L‐glutamate, pyruvate, and succinate (Angélica et al., 2014). D‐Leucine and L‐valine are branched chain amino acids (BCAAs), especially leucine, and can enhance protein synthesis through the mTOR signaling pathway to regulate energy balance, which plays a vital role in blastocyst development. Banerjee et al. found that the metabolites, including lysine, L‐arginine, glutamine, threonine, histidine, phenylalanine, and tyrosine, were significantly increased in patients with idiopathic recurrent spontaneous miscarriage, which may be involved in vascular dysfunction associated with poor endometrial receptivity and excessive inflammatory reactions (Priyanka et al., 2014). Houghton et al. used reversed‐phase high‐performance liquid chromatography (RHPLC) and found that the content of leucine was significantly reduced after in vitro fertilization when the embryo developed into the cyst embryo culture medium, while the contents of valine and isoleucine were significantly reduced in the embryo culture medium that did not develop into the blastocyst. Valine and isoleucine have a definite influence on embryo development (Houghton et al., 2002). Zhang et al. found that BCAAs are closely involved in pregnancy outcome. The elevated BCAAs can clearly impair the development of diploids cells to the morula and blastocyst stages (Thuan et al., 2002). Xiao‐yao Miao et al. used metabolomic‐based gas chromatography‐mass spectrometry and pattern recognition to study the potential mechanism of Danggui Buxue Tang's anti‐fatigue effect on weight‐bearing forced swimming mice and found that Danggui Buxue Tang had a good anti‐fatigue effect (Miao et al., 2018). In our study, we found that the glycine, D‐leucine, and L‐valine serum concentrations in the AWE group were higher than those in the M group, which may indicate that *Angelica sinensis* may affect the inflammatory process in AUB rats.

Lipid metabolism mainly consists of linoleic acid, glycerophospholipid, and sphingolipid metabolism. Oleic acid, which is closely related to leukotriene synthesis, is considered to be a key factor in tissue injury, inflammation, and vasoconstriction. Kim et al. found that the water extract of *Angelica sinensis* has an anti‐inflammatory effect via the NO‐burst/calcium‐mediated JAK‐STAT pathway (Young‐Jin et al., 2018). Yao et al. found that the anti‐inflammatory activity of the volatile oil of *Angelica sinensis* mainly through regulating glycine and arachidonic acid metabolic disorders (Yao et al., 2015). And in our study, after AWE intervention, the AWE group showed significant upregulation of N‐Acetyl‐leukotriene E4, 20‐Oxo‐leukotriene E4, and Leukotriene D5, which indicated that the effect of AWE on rats with AUB may involve the regulation of arachidonic acid metabolic network disorders. Lyso PC, a phospholipid, can regulate vascular tone and induce endothelial dysfunction. It is reported that saturated fatty acids can induce the expression of cyclooxygenase (Akito et al., 2009) and promote the synthesis of PGF2α (Helliwell et al., ). In this study, LysoPC (16:0), LysoPC (17:0), PC (18:1(9Z)/18:1(9Z)), LysoPC (18:2), LysoPC (18:3), LysoPC (20:5), and LysoPC (18:0/0:00) were up‐regulated in the AWE group. These results indicate that glycerophospholipid metabolism was abnormal.

Sphinganine is a sphingolipid that is involved in the formation of cell membranes. It can be phosphorylated under the catalysis of sphingosine kinase to produce a potent signaling lipid molecule, sphingosine‐1‐phosphate (S1P). S1P can regulate the physiological functions of cell survival, growth, proliferation, and apoptosis from the extracellular receptor pathway and the intracellular second messengers. Roth et al. indicated that sphingosine‐1‐phosphate can promote the maturation of bovine oocytes and enhance the development of embryos (Roth & Hansen, 2004). Hannoun et al. indicated that the fragmentation rate of human preimplantation embryos medium with S1P was significantly lower and the embryos quality was better (Antoine et al., 2010). In our study, we found that the serum concentration of sphingosine in the AWE group was higher than that in the M group, which may be because AWE has a regulatory effect on sphingosine.

All‐trans‐retinoic acid belongs to vitamin A (retinol), which is a basic nutrient required for mammalian reproduction. This molecule plays a vital role in promoting the normal development of embryos, regulating cell proliferation and differentiation, and maintaining normal cell differentiation and immune system function integrity. Ueda et al. showed that chemical signaling induced by the use of retinoic acid can promote the differentiation of embryonic stem cells into neurons (Ueda et al., 2018). In our study, we found that the level of all‐trans‐retinoic acid was down‐regulated in the AWE group. In summary, AWE could adjust the abnormal metabolism state of amino acid, retinol, and lipid metabolism with AUB induced by incomplete medical abortion.

## 5 CONCLUSIONS

An UPLC‐Q‐TOF‐MS‐based serum metabolomic approach was applied to investigate the mechanisms of AWE for the treatment of AUB. Twenty‐one potential biomarkers were eventually identified, which may be involved in the intervention mechanism of AWE for the treatment of AUB. Some metabolic pathways including primary bile acid biosynthesis, glycerophospholipid metabolism, pentose and glucuronate interconversions, glutathione metabolism, glyoxylate and dicarboxylate metabolism, sphingolipid metabolism, glycine, serine and threonine metabolism, retinol metabolism, ascorbate, and aldarate metabolism were altered by AWE treatment. The significantly reversed the metabolic aberrations in AUB group by AWE facilitates to support the therapeutic effect and potential mechanisms of AWE on AUB induced by incomplete medical abortion.

## CONFLICT OF INTEREST

The authors declare no conflict of interest.

## Data Availability

The data that support the findings of this study are available from the corresponding author upon reasonable request.
